# Isotretinoin as a Possible Cause for Eosinophilic Esophagitis and Eosinophilic Gastroenteritis: A Case Report

**DOI:** 10.7759/cureus.42649

**Published:** 2023-07-29

**Authors:** Anjala Nizam, Sarah Gaballah, Labib Al Ozaibi, Lakshmiah G Raman

**Affiliations:** 1 Department of Medicine and Surgery, Dubai Academic Health Corporation, Dubai, ARE; 2 Department of General Surgery, Rashid Hospital, Dubai, ARE; 3 Department of Pathology and Genetics, Dubai Hospital, Dubai, ARE

**Keywords:** eosinophilia, eosinophilic gastrointestinal disorders, isotretinoin, eosinophilic gastroenteritis, eosinophilic esophagitis

## Abstract

A 39-year-old female patient who took isotretinoin for severe acne for around six months presented with severe upper abdominal pain, as well as abdominal distention. Initially, she was diagnosed with acute cholecystitis due to the presence of gallstones on ultrasound. However, additional imaging showed mural thickening of the bowel, for which she underwent further work-up. Laboratory investigations showed raised inflammatory markers along with eosinophilia. Concurrently, bedside paracentesis showed raised levels of eosinophils. The patient underwent an endoscopic assessment, which revealed eosinophilic esophagitis and gastroenteritis likely to be induced by isotretinoin. Following the discontinuation of isotretinoin and the initiation of corticosteroid therapy, the patient's clinical condition improved significantly. The diagnosis of eosinophilic gastrointestinal disorders, though rare, must be kept in mind when patients on long-term isotretinoin treatment or with other risk factors present with symptoms such as dysphagia, abdominal pain, odynophagia, nausea, and vomiting.

## Introduction

Eosinophilic gastroenteritis (EGE) and eosinophilic esophagitis (EoE) are diseases that fall under the umbrella of “eosinophilic gastrointestinal disorders” (EGDs). It refers to a range of diseases that are considered to be rare and that affect segments of the gastrointestinal (GI) tract with eosinophilic inflammation, without secondary causes [1]. Orally administered isotretinoin is a systemic retinoid that affects sebaceous glands and is used to treat severe nodular acne that is resistant to conventional treatment, including systemic antibiotics. It has several side effects such as cheilitis, xerosis, xerostomia, dry nose, skin sensitivity, and hypertriglyceridemia [2]. However, despite an extensive literature review, no cases of isotretinoin-induced EoE or gastroenteritis have been reported. Hence, here we present a unique case of EoE and EGE likely due to the concomitant use of isotretinoin.

## Case presentation

A 39-year-old female known patient of hypothyroidism on levothyroxine and acne vulgaris for which she was taking isotretinoin for around six months presented to the emergency department (ED) with a three-day history of severe upper abdominal pain. The pain was associated with a two-week history of abdominal distention and a six-week history of dysuria and perianal itching; however, the patient denied any nausea, vomiting, and altered bowel habits. She presented with the same complaints to her family physician a few weeks prior to presenting to the ED. At the time, omega 3 was prescribed, but her symptoms persisted; therefore, isotretinoin was discontinued 10-15 days prior to presentation. Furthermore, she had recently developed body aches and bilateral foot pain, which were diagnosed as isotretinoin-induced myalgia and plantar fasciitis. As a result of the isotretinoin treatment, she also developed hypertriglyceridemia. The systemic review was otherwise unremarkable for atopy or extraintestinal manifestations. Besides having hypothyroidism and acne vulgaris, her past medical and surgical history includes an abdominoplasty and a road traffic accident that resulted in lung damage, arm fractures, and rib fractures. She received the last of her two doses of the Pfizer-BioNTech COVID-19 vaccine one year prior to presentation.

**Figure 1:**
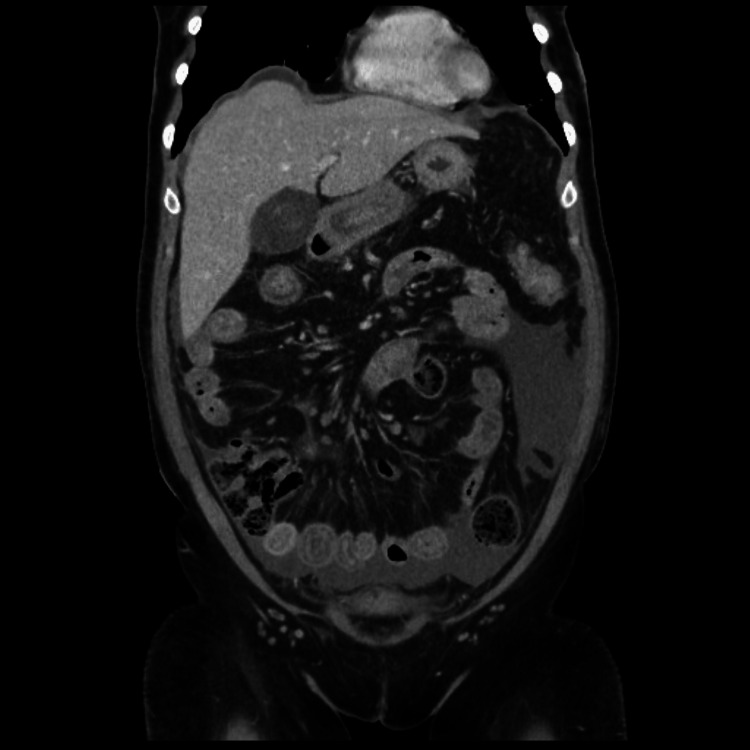
Computed tomography scan of the abdomen showing mural thickening of the small and large bowel.

The bedside ultrasound imaging performed in the ED showed cholelithiasis and ascites. Hence, she was initially admitted under the care of the general surgery team as a case of acute abdomen. Further radiological evaluation confirmed the presence of cholelithiasis, but there were no signs of acute cholecystitis. A contrast-enhanced computed tomography (CT) scan demonstrated mural thickening of the small and large bowel (Figure 1).

Her blood investigations revealed marked leukocytosis with a white blood cell count of 18.7x10^3^/uL and an absolute eosinophil count of 8.1x10^3^/uL, mildly elevated inflammatory markers, and elevated total immunoglobulin E (IgE) levels (Table 1).

**Table 1:** Laboratory investigations done at the time of admission to the hospital WBC, white blood cell; RBC, red blood cell

Laboratory investigation	Laboratory value	Normal reference range
Complete blood count		
WBC count	18.7 k/uL	3.6-11 10^3^/uL
RBC count	4.71 MIL/uL	3.80-4.80 10^6^/uL
Hemoglobin, blood	13.0 g/dL	12-15 g/dL
Hematocrit	39.9 %	36%-46%
Platelet count	347 k/uL	150-410 k/uL
Differential count		
Nucleated RBCs	0 per100WBC	0 per100WBC
Neutrophils absolute	6.9 x 10^3^/uL	2.0-7.0 x 10^3^/uL
Lymphocytes absolute	3.0 x 10^3^/uL	1.0-3.0 x 10^3^/uL
Monocytes absolute	0.7 x 10^3^/uL	0.2-1.0 x 10^3^/uL
Eosinophils absolute	8.1 x 10^3^/uL	0.0-0.5 x 10^3^/uL
Basophils absolute	0.0 x 10^3^/uL	0.0-0.1 x 10^3^/uL
Inflammatory markers		
C-reactive protein	14.5 mg/L	<5.0 mg/L
Procalcitonin	0.08 ng/mL	<0.05 ng/mL
Immunoglobulins		
Immunoglobulin G	12.4 g/L	7.0-16.0 g/L
Immunoglobulin A	2.16 g/L	0.7-4.0 g/L
Immunoglobulin M	1.51 g/L	0.4-2.3 g/L
Immunoglobulin E (Total)	385.0 kU/L	<100 kU/L: negative; >100 kU/L: 78% atopic

She completed a course of cefuroxime and analgesics with no improvement in her clinical status; thus, she was started on meropenem, which also did not improve her condition. The gastroenterology team was then involved in treatment, and a bedside abdominal paracentesis was performed, which showed a very high total nucleated cell count of 3,508/mm^3^ with eosinophils forming 98% of it (Table 2).

**Table 2:** Peritoneal fluid cell count

Laboratory investigation	Laboratory value	Normal reference range
Total nucleated cells	3508 cells/mm^3^	<500 cells/mm^3^
Differential count		
Neutrophils	0%	<25%
Lymphocytes	0%	<75%
Monocytes	2%	<1%
Eosinophils	98%	<1%

**Figure 2:**
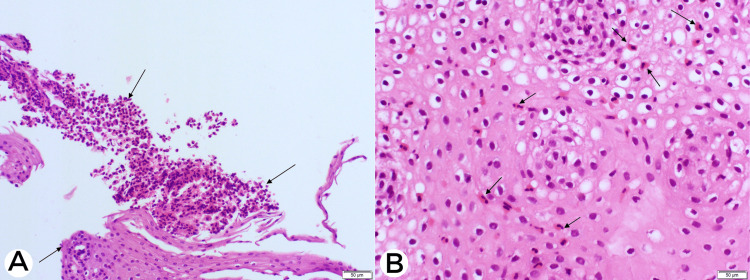
Esophageal mucosa with H&E stain in 200x view showing (A) intraepithelial eosinophils (black arrows) and (B) surface involvement by eosinophils (black arrows). H&E, hematoxylin and eosin

She then underwent endoscopic assessment with esophagogastroduodenoscopy and colonoscopy, followed by capsule endoscopy, which revealed mild erosive gastritis with ulcerations in the small bowel but a normal colorectal mucosa. The esophagus was mildly dilated, and the Z-line was found to be irregular at 37 cm from the incisors. There were no signs suggestive of EoE. Biopsies were taken from the esophagus, stomach, duodenum, and colon. Considering the above findings, the patient was then evaluated for the following possible etiologies such as vasculitis, malignancy, parasitic infection, and tuberculosis. However, the workup for the aforementioned etiologies turned out to be negative. In addition, stool analysis conducted on multiple occasions did not reveal any parasites. The histopathological analysis of the biopsies taken during the endoscopic assessment revealed mucosal EoE (more than 20/hpf), eosinophilic gastritis (more than 20/hpf), eosinophilic duodenitis (more than 30/hpf), and eosinophilic colitis (30/hpf). (Figure 2).

It was then concluded that the patient had mucosal EoE and EGE likely to be induced by drug. A dramatic clinical improvement was seen within 24 hours after she was put on hydrocortisone, and the antibiotics were stopped. It was also associated with a decrease in the level of peripheral eosinophilia. She was then discharged on a tapering course of oral budesonide. During her one- and six-month post-discharge follow-up visits, she reported a complete resolution of her symptoms.

## Discussion

There are a number of EGDs including the two disorders discussed in this case report, i.e., EoE and EGE. EGDs are characterized by eosinophilic infiltration of the GI tract. In addition to dysphagia and vomiting, EGDs can also cause food impaction in the esophagus, abdominal pain, diarrhea, weight loss, and malabsorption-related signs, including iron deficiency and losing protein enteropathy. In rare cases, there can be extensive infiltration of eosinophils reaching the serosa leading to eosinophilic ascites [3].

There are three forms of the disease depending on the extent of digestive tissue involvement: mucosal (45-80%), muscular (12-30%), and serosal (12.5-39%) [4]. The pathophysiology of the disease is not yet completely understood; however, genetics and environment play an important role in triggering T helper 2 (Th2) allergic reactions and inflammation, leading to diminishing the epithelial barrier integrity and damaging the mucosa [5,6].

Clinical symptoms and signs of EGDs are determined by what part of the GI tract is affected. Endoscopy and histopathology sample is the gold standard, but there is no established cut-off agreed upon with regard to eosinophilic count for most EGDs, unlike EoE, where an internationally accepted diagnostic cut-off point of 15/hpf has been established; however, EGE has no such threshold, and the most commonly accepted cut-off is 20/hpf [7].

Caution should be taken before labeling a patient with EGDs, as other diseases, for example, inflammatory bowel diseases, autoimmune diseases, infections, and lymphoproliferative malignancies, are associated with a similar clinical picture and peripheral eosinophilia. When distinguishing them from EGDs, a holistic approach must be taken, starting with clinical presentation, laboratory findings, endoscopy, and histopathology biopsies [3].

There are no established causes recognized for EGDs; however, some risk factors may include hypersensitivity reaction to a certain food, medications (such as gold salts, azathioprine, gemfibrozil, trimethoprim-sulfonamide, enalapril, carbamazepine, and nivolumab), and a family history of allergic disorders (such as asthma, hay fever, or eczema) [8-14].

No cases of isotretinoin-induced EGDs have been reported despite a thorough literature search; however, there exist two case reports on isotretinoin-induced esophagitis and/or esophageal ulcers. In the first case, the patient presented with dysphagia, which rapidly improved after the cessation of isotretinoin [15]. The second patient developed severe, progressive odynophagia after taking isotretinoin for three weeks. After the exclusion of cytomegalovirus (CMV), human immunodeficiency virus (HIV), and herpes simplex virus (HSV), a biopsy result of esophagitis along with a temporal relationship to the initiation of isotretinoin, the diagnosis of isotretinoin-induced esophagitis was made [16]. The third case is of a patient who presented with severe odynophagia following one month of isotretinoin use. Several esophageal ulcers were noticed on endoscopic assessment, possibly secondary to isotretinoin use. The symptoms disappeared upon stopping the isotretinoin pills and initiating paracetamol and sucralfate liquid therapy [17]. Patients taking isotretinoin should be clearly instructed about taking the pill with copious water, and patients presenting with odynophagia or dysphagia should be screened for esophagitis and EoE.

A case of COVID-19 vaccine-associated EGD was reported in South Korea where a patient developed abdominal pain, abdominal distention, nausea, diarrhea, and dyspnea immediately after receiving the second dose of the Pfizer-BioNTech COVID-19 mRNA vaccine. Subserosal EGE, which presented as isolated eosinophilic ascites, was demonstrated on endoscopic assessment. The patient made a rapid recovery after receiving prednisolone [18]. The patient mentioned in our case report too had taken the Pfizer-BioNTech COVID-19 vaccine. However, her last dose of the COVID-19 vaccine was over one year prior to the development of her symptoms, which makes it unlikely to be the cause of her EGD diagnosis.

The mainstay of treatment for EoE and EGE is systemic steroids. In most cases, patients report rapid improvements in their condition after they start using oral corticosteroids.

## Conclusions

EGDs in the past few decades were not a field of interest for gastroenterologists due to the rarity of the disease; however, there is wide recognition of EoE as it is the most commonly diagnosed disease within this family. This is the first report suggesting a possible association between isotretinoin and EGD. Further research is needed to conclude isotretinoin use as an established cause of EGDs.
